# Delay of appropriate antibiotic treatment is associated with high mortality in patients with community-onset sepsis in a Swedish setting

**DOI:** 10.1007/s10096-019-03529-8

**Published:** 2019-03-25

**Authors:** Maria Andersson, Åse Östholm-Balkhed, Mats Fredrikson, Martin Holmbom, Anita Hällgren, Sören Berg, Håkan Hanberger

**Affiliations:** 1 Division of Infectious Diseases, Department of Clinical and Experimental Medicine, Faculty of Medicine and Health Sciences, Linköping University, Linköping, Sweden; 2 Occupational and Environmental Medicine, Department of Clinical and Experimental Medicine, Faculty of Medicine and Health Sciences, Linköping University, Linköping, Sweden; 3 Forum Östergötland, Faculty of Medicine and Health Sciences, Linköping University, Linköping, Sweden; 4 Department of Urology and Department of Clinical and Experimental Medicine, Linköping University, Linköping, Sweden; 5 Division of Cardiothoracic Anesthesia and Intensive Care, Department of Medicine and Health Science, Faculty of Medicine and Health Sciences, Linköping University, Linköping, Sweden

**Keywords:** Sepsis, Septic shock, Antibiotics, Mortality, Emergency department

## Abstract

Early appropriate antimicrobial therapy is crucial in patients with sepsis and septic shock. Studies often focus on time to first dose of appropriate antibiotics, but subsequent dosing is equally important. Our aim was to investigate the impact of fulfillment of early treatment, with focus on appropriate administration of first and second doses of antibiotics, on 28-day mortality in patients with community-onset severe sepsis and septic shock. A retrospective study on adult patients admitted to the emergency department with community-onset sepsis and septic shock was conducted 2012–2013. The criterion “early appropriate antibiotic treatment” was defined as administration of the first dose of adequate antibiotics within 1 h, and the second dose given with less than 25% delay after the recommended dose interval. A high-risk patient was defined as a septic patient with either shock within 24 h after arrival or red triage level on admittance according to the Medical Emergency Triage and Treatment System Adult. Primary endpoint was 28-day mortality. Of 90 patients, less than one in four (20/87) received early appropriate antibiotic treatment, and only one in three (15/44) of the high-risk patients. The univariate analysis showed a more than threefold higher mortality among high-risk patients not receiving early appropriate antibiotic treatment. Multivariable analysis identified early non-appropriate antibiotic treatment as an independent predictor of mortality with an odds ratio for mortality of 10.4. Despite that the importance of early antibiotic treatment has been established for decades, adherence to this principle was very poor.

**Electronic supplementary material:**

The online version of this article (10.1007/s10096-019-03529-8) contains supplementary material, which is available to authorized users.

## Introduction

Sepsis and septic shock are common causes of mortality and morbidity in Sweden and globally. The reported annual incidence of sepsis varies depending on the method used to identify cases, but estimates of sepsis and septic shock rates lie around 500/100,000/year and 30–50/100,000/year, respectively [1–7].

Early recognition with resuscitation [8] and appropriate antibiotic treatment is known to be crucial for survival. Delayed appropriate antibiotic treatment has been shown to be an independent risk factor for mortality both in severe sepsis and septic shock [9–15], a fact that was confirmed in a recent systematic review of the literature [16].

Time to first antibiotic has been well studied, but administration of subsequent doses has received less attention. However, Leisman et al. [17] found that a major delay (> 25%) of the second dose was associated with increased hospital mortality. This is supported by pharmacokinetic/pharmacodynamic (PK/PD) studies showing that optimising antibiotic administration may increase survival [18–20].

The Surviving Sepsis Campaign Guidelines recommends broad-spectrum antibiotics within 1 h after detection as a part of the treatment bundle [21]. We are unaware of any treatment bundle including subsequent dosing. Consequently, less attention is paid to improving this in sepsis quality initiatives.

Our hypothesis was that early appropriate antibiotic treatment, i.e. first and second antibiotic dose at the right time, leads to improved outcome.

The aim of this study was to investigate the effect of early treatment on 28-day mortality, with focus on appropriate administration of first and second doses of antibiotic(s), in patients with community-onset severe sepsis and septic shock.

## Materials and methods

### Study design and settings

**Fig. 1:**
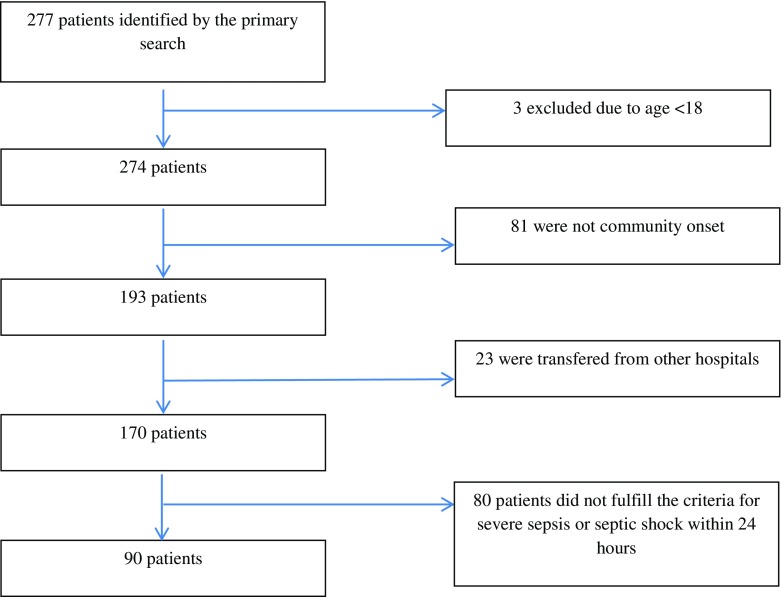
Flowchart for inclusion and exclusion

A retrospective, observational cohort study was conducted at Linköping University Hospital, Sweden, from September 30, 2012, until September 30, 2013. All patients aged 18 years or older with the International Classification of Disease ICD-10SE diagnosis A 40 (bacteraemia caused by Streptococci), A 41 (other bacteraemia), R65.1 (severe sepsis) or R57.2 (septic shock) [22] at discharge, cared for in the intensive care unit (ICU) and the Departments of Infectious Disease and Acute Internal Medicine including the medical high dependency unit, were identified. We included only patients admitted to these departments since they are specialised in caring for patients with community-onset severe sepsis or septic shock. Of these, patients who met the criteria of community-onset (defined as no hospitalisation within 30 days prior to admission) severe sepsis or septic shock within 24 h after arrival at the emergency department (ED) were included. Flowchart for inclusion and exclusion is shown in Fig. 1.

The study was performed in a tertiary care university hospital which serves a primary population of 172,000, with no other ED in the catchment area. Patients transferred from another hospital were excluded.

Patients were only included once, i.e. the primary admission with community-onset severe sepsis or septic shock within the study period.

### Data collection

All data and records were reviewed by the first author. Any ambiguities concerning application of definitions or clinical treatments were resolved within the study group consisting of senior infectious disease consultants and an ICU physician. The primary endpoint was 28-day mortality. Secondary outcomes were achievement of “early appropriate antibiotic treatment” and treatment goals at 1 and 6 h according to the Surviving Sepsis Campaign (SSC) at the time, and the Swedish Society of Infectious Diseases’ treatment goals [23, 24].

The following baseline characteristics were registered: gender, age, weight, renal function (eGFR < 30 ml/min × m^2^) and previously diagnosed comorbidity. Severity of disease was assessed by Medical Emergency Triage and Treatment System Adult (METTS) [25, 26], APACHE II score [27] and SOFA score [28]. Indicators of appropriate early treatment (resuscitation, antibiotic treatment and fulfilment of early treatment goals (see definition below)), limitation of level of care, ICU admission, length of stay, indicators of organ dysfunction, focus of infection, aetiology and 28-day mortality were also registered.

APACHE II and SOFA scores were calculated from the time of arrival at the emergency department. Laboratory parameters and vital signs were followed over the first 48 h (exception made for CRP which was followed until 72 h after admission).

Antibiotic administration data recorded were as follows: agent(s), dose, time of administration of first and second doses of appropriate antibiotic and recommended dose interval(s).

### Definitions of appropriate and early antibiotic treatment

1. Appropriate antibiotic treatment: The following major criteria had to be fulfilled:

- Treatment with antibiotic agent(s), including dose and dose interval according to the national treatment guidelines [24]
- Susceptibility of the pathogen(s) isolated to the agent(s) used (if the patient had a culture-negative sepsis only “a” had to be fulfilled) [11]

2. Early appropriate antibiotic treatment

To fulfil the definition of early appropriate antibiotic treatment, the 1st dose fulfilling the major criteria had to be administered within 1 h, and the 2nd dose fulfilling the major criteria given with less than 25% delay based on the recommended dose interval in the national treatment guidelines.

In cases of uncertainty, evaluation of the appropriateness of antibiotic treatment was discussed in the study group consisting of senior infectious disease consultants and an ICU physician.

The timing of the 2nd dose of an appropriate antibiotic with renal elimination took into consideration the increased dose interval due to reduced renal excretion. When more than one adequate antibiotic was given, the longest recommended dose interval was applied [17]. The ratio for time interval between the first and second doses was calculated as the actual time divided by the recommended time interval. For patients not receiving effective antibiotic therapy within 24 h, time to second dose and 1st-to-2nd dose interval ratio was not calculated.

### Treatment goals and resuscitation

Treatment goals for the first hour were systolic blood pressure > 90 mmHg and oxygen saturation > 93%. Goals within 6 h were urinary production > 0.5 ml/kg/h, fall in lactate level and mean arterial pressure (MAP) ≥ 65 mmHg [23, 24].

Fluid resuscitation was recorded as type and amount of fluid given in the time intervals 0–2 h, 2–6 h and 6–24 h. Since the composition of the fluid therapy (crystalloids, colloids and blood products) varied between patients, the predicted intravascular volume expansion was calculated in order to compare the effect of fluids administered. Taking into account their extravascular distribution, crystalloid volume was multiplied by a factor of 0. 25, albumin solutions 40–50 mg/ml by a factor of 0.5, albumin solutions 200 mg/ml by a factor of 1.5, plasma by a factor of 0.5 and blood transfusion by a factor of 1. Glucose infusion given slowly for calorific purposes was not included in the fluid calculations.

### Limitation of level of care

For some of the patients included, the decision was made to limit the level of care. As such a decision influences both treatment given and mortality, this was a potential confounding factor. Limitation of level of care set within the first 24 h was registered as withholding: (a) ICU, (b) ventilator support, (c) dialysis, (d) cardiopulmonary resuscitation (CPR) and (e) withdrawal of all active treatment.

### Definitions of sepsis, high-risk patient, infection and focus of infection

This study was performed prior to the introduction of the new sepsis definitions, Sepsis-3 [29], and hence, definitions according to the SSC criteria of 2012 were used [23].

*Sepsis:* The systemic inflammatory response (SIRS) to infection [30].

*Severe sepsis*: Sepsis with hypotension (systolic blood pressure (SBP) < 90 mmHg or MAP < 70 mmHg), hypoperfusion (P-lactate > 1 mmol/l above reference (3.4 mmol/l), base excess (BE) ≤ − 5 mmol/l) or one or more of the following organ dysfunctions: renal (oliguria < 0.5 ml/kg/h ≥ 2 h despite resuscitation, or serum creatinine elevated > 45 μmol above the patient’s former value if known), respiratory (PaO_2_/FiO_2_ < 33 kPa without or < 27 kPa with pneumonia), coagulation defect (platelet count < 100 × 10^9^/l, PT-INR > 1.5 or APTT > 60s),CNS (altered mental status) or hepatic (S-bilirubin > 45 μmol/l).

*Septic shock:* Severe sepsis with sepsis-induced hypotension unresponsive to fluid resuscitation, together with hypoperfusion or organ dysfunction [3, 23, 30].

*High-risk patient:* Patients with red triage level according to METTS on arrival at the ED, or patients with septic shock on admission or shock within 24 h after arrival at the ED. Red triage level according to METTS is defined as a life-threatening condition requiring immediate medical attention by a physician. This identifies a more severely ill cohort of severe sepsis patients where more prompt treatment on the ED might have prevented progress to shock. Thus, the combination resulting in the high-risk patient definition better identifies the population of severely ill septic patients.

*Infection sites:* The definitions suggested by the International Sepsis Forum Consensus Conference on Definitions of Infections in the Intensive Care Unit [31] were used, with minor changes.

*Bloodstream infection (BSI)*: Isolation of bacteria or fungi from at least one blood culture. Microorganisms typically belonging to skin flora were considered a contamination.

*Pneumonia:* Symptoms and radiology indicating pneumonia.

*Urosepsis:* Positive culture from blood or two of the following: fever >38 °C, symptoms of UTI, relevant pathogens in the urine or radiographic evidence.

*Skin and soft tissue infection:* Erysipelas, cellulitis, fasciitis or wound infection.

*Intra-abdominal infection:* Symptoms and radiologic evidence, or findings during surgery of intra-abdominal infection. In case of surgery, positive blood cultures were required prior to surgery.

*Infective endocarditis:* Definite infective endocarditis according to the modified Duke criteria [31, 32].

*Microbiological aetiology:* Detection of relevant pathogens in cultures, by antigen testing or PCR technique.

*Community-onset:* No hospitalisation within 30 days prior to admission.

### Statistical analyses

The primary outcome variable was 28-day mortality. The whole study group was analysed as such, and a subgroup analysis of high-risk patients was made. The statistical programs STATA v15 and SPSS version 24 were used for the analyses. We compared normally distributed quantitative variables with the *t* test and categorical variables using the chi^2^ test or Fisher exact test. Mann–Whitney *U* test was used for fluid volumes. Double-sided *P* values were used. A *P* value < 0.05 was considered statistically significant. Missing data were treated by pairwise deletion, and there was no imputation. A multivariable analysis was performed with logistic regression including the univariate parameters correlating to 28-day mortality followed by stepwise removal of parameters.

## Results

### Characteristics of the study population

There were 90 patients enrolled in the study, mean age was 73 years and 51% were female. Overall 28-day mortality was 26.7%. Baseline characteristics are shown in Table 1. Sixty-three patients had severe sepsis without shock and 27 patients developed shock within 24 h, with mortality rates of 17.5% and 48.2%, respectively. There were no differences in age or gender regarding the risk for developing septic shock (data not shown).

**Table 1:** Baseline characteristics of survivors and non-survivors

	Total study population			High-risk patients		
	Survivors (*n* = 66)	Non-survivors (*n* = 24)	*P* value	Survivors (*n* = 29)	Non-survivors (*n* = 17)	*P* value
Age (years) (SD)	70 (15)	83 (9)	*< 0.001*	68 (15)	83 (11)	*0.001*
Female sex (%)	35 (53)	11 (45.8)	0.546	15 (51.7)	8 (47.1)	0.76
Preexisting comorbidity (%)						
Malignancy, all	6 (9.1)	5 (20.8)	0.154	3 (10.3)	4 (23.5)	0.397
Metastatic malignancy	1 (1.5)	3 (12.5)	0.056	1 (3.4)	2 (11.8)	0.545
Haematological malignancy	3 (4.5)	1 (4.2)	0.999	2 (6.9)	1 (5.9)	0.999
Diabetes mellitus	14 (21.2)	6 (25)	0.702	5 (17.2)	4 (23.5)	0.707
Congestive heart failure	8 (12.1)	6 (25)	0.187	2 (6.9)	4 (23.5)	0.174
Immunosuppression, any	19 (28.8)	5 (20.8)	0.450	10 (37.9)	3 (17.6)	0.315
Immunosuppression, > 10 mg Prednisolone	9 (13.6)	0	0.106	4 (13.8)	0	0.281
Chronic pulmonary disease	8 (12.1)	5 (20.8)	0.320	4 (13.8)	4 (23.5)	0.443
Chronic renal failure (GFR < 30)	4 (6.1)	1 (4.2)	0.999	1 (3.4)	0	0.999
Severity of disease						
APACHE II (SD)	19.0 (5.7)	21.4 (7.1)	0.101	21.5 (4.8)	23.6 (7.0)	0.235
Maximum SOFA (SD)	5.2 (3.0)	6.3 (3.7)	0.132	6.2 (3.4)	7.4 (3.8)	0.265
SBP < 90 mmHg/MAP < 65 mmHg (SD)	57 (86.4)	19 (79.2)	0.511	25 (86.2)	15 (88.2)	0.999
Septic shock (%)	14 (21.2)	13 (54.2)	*0.003*	14 (48.3)	13 (76.5)	0.061
High-risk patients (%)	29 (43.9)	17 (70.8)	*0.024*	29 (100)	17 (100)	
Maximum lactate level day 1 (SD)	2.4 (1.7) *n* = 53	4.4 (2.8) *n* = 23	*< 0.001*	2.7 (1.7) *n* = 28	4.9 (2.9) *n* = 17	*0.003*
Number of organ dysfunction (SD)	1.35 (1.0)	2.21 (1.4)	*0.002*	1.7 (1.1)	2.8 (1.3)	*0.006*
Respiratory (%)	19 (28.8)	14 (58.3)	*0.010*	13 (44.8)	14 (82.4)	*0.013*
Renal (%)	37 (56.1)	20 (83.3)	*0.018*	20 (69.0)	15 (88.2)	0.172
Haematological/coagulopathy (%)	14 (21.2)	8 (33.3)	0.237	9 (31.0)	8 (47.1)	0.277
CNS (%)	15 (22.7)	9 (37.5)	0.161	8 (27.6)	8 (47.1)	0.181
Liver (%)	2 (3.0)	2 (8.3)	0.288	0	2 (11.8)	0.131

Data are presented as no. (%) or mean (SD) as indicated. The total study population and the high-risk patients are analysed separately. *t* Test, Pearson chi^2^ or Fisher’s exact test, as appropriate. *P* values < 0.05 are shown in italics

Significantly increased mortality in the total study population was associated with higher age, presence of shock or being a high-risk patient (see below), higher maximum lactate level, respiratory and renal dysfunction due to the infection and number of dysfunctional organs.

A subgroup analysis was made on high-risk patients (patient with red triage level according to METTS in the ED or shock within 24 h after arrival at the ED) (Table 1).

Clinical interventions and treatment evaluations are shown in Table 2. Limitation of level of care and failure to achieve haemodynamic stability within 6 h (MAP ≥ 65 mmHg within 6 h) were associated with increased mortality. More extensive fluid administration late in the course (6–24 h) was also related to higher mortality. Twenty-four patients were treated for sepsis on the ICU at some time during their hospital stay. The 28-day mortality rate was 38% among ICU-treated patients (9/24) and 23% (15/66) among patients treated on the general ward or intermediate care unit. This difference, however, was not significant.

**Table 2:** Clinical interventions and treatment evaluation among survivors and non-survivors

	Total study population			High-risk patients		
	Survivors (*n* = 66)	Non-survivors (*n* = 24)	*P* value	Survivors (*n* = 29)	Non-survivors (*n* = 17)	*P* value
Volume (ml), median (IQR)						
Crystalloids 0–6 h	2000 (2000–3000)	2000 (1500–3500)	0.888	3000 (2000–5000)	3000 (2000–4000)	0.430
Plasma expansion 0–6 h	500 (500–900)	515 (500–1025)	0.540	875 (500–1375)	1000 (500–1150)	0.839
Crystalloids 6–24 h	2000 (1000–3000)	2200 (1000–3000)	0.381	2000 (1000–4000)	3000 (2000–4000)	0.230
Plasma expansion 6–24 h	500 (250–910)	842 (500–1337)	*0.027*	625 (450–1230)	1100 (705–1875)	*0.040*
Appropriate antibiotics within 1 h (%)	19 (29.2) *n* = 65	5 (22.7) *n* = 22	0.555	15 (53.6) *n* = 28	4 (25.0) *n* = 16	0.066
2nd dose without > 25% delay (%)	48 (84.2) *n* = 57	18 (81.8) *n* = 22	0.784	22 (84.6) *n* = 26)	13 (81.3) *n* = 16	0.999
Early appropriate antibiotic treatment (%)	17 (26.2) *n* = 65	3 (13.6) *n* = 22	0.228	13 (46.4) *n* = 28	2 (12.5) *n* = 16	*0.022*
ICU admission (%)	15 (22.7)	9 (37.5)	0.161	10 (34.5)	7 (41.2)	0.650
ICU admission from ED (%)	5 (7.6)	5 (20.8)	0.123	5 (17.2)	4 (23.5)	0.707
Limits of level of care (%)	10 (15.2)	13 (54.2)	*< 0.001*	4 (13.8)	12 (70.6)	*< 0.001*
Within 1 h: SBP > 90 mmHg (%)	41 (75.9) *n* = 54	12 (57.1) *n* = 21	0.109	15 (57.7) *n* = 26	6 (40.0) *n* = 15	0.275
Within 1 h: saturation > 93% (%)	42 (82.4) *n* = 51	12 (60.0) *n* = 20	0.065	19 (76.0) *n* = 25	8 (57.1) *n* = 14	0.287
Within 6 h: urinary production > 0.5 ml/kg (%)	15 (53.6) *n* = 28	6 (60.0) *n* = 10	0.999	7 (46.7) *n* = 15	4 (50) *n* = 8	0.999
Within 6 h: lowered lactate (%)	9 (60.0) *n* = 15	6 (46.2) *n* = 13	0.464	6 (60) *n* = 10	6 (50) *n* = 12	0.691
Within 6 h: MAP ≥ 65 mmHg (%)	31 (53.4) *n* = 58	5 (23.8) *n* = 21	*0.019*	12 (48.0) *n* = 25	2 (12.5) *n* = 16	*0.019*

Data are presented as no. (%) or mean (SD) unless otherwise indicated. The total study population and the high risk patients are analysed separately. *t* Test, Pearson chi^2^ or Fisher’s exact test, as appropriate. Volume calculated with Mann–Withney *U*. *P* values < 0.05 are shown in italics

*SBP* systolic blood pressure, *MAP* mean arterial pressure, *BE* base excess, *SD* standard deviation, *ICU* intensive care unit, *SOFA* sequential organ failure assessment, *APACHE* acute physiology and chronic health evaluation, *ED* emergency department

During the first 24 h, 26% (23/90) of the patients had their level of care limited, and their mortality rate was 56.5%. Typical for this group were increased age, presence of congestive heart failure, higher APACHE II score and an increased number of failing organs due to infection. These patients received more fluid between 6 and 24 h, but there were no other significant differences regarding clinical interventions. Data on this are presented in the supplementary files. When excluding patients with care limitation, mortality in the study population was 16% (11/67) and in the high-risk group 17% (5/30).

### Focus of infection, microbiological diagnosis

Urinary tract infection (UTI) followed by pneumonia, skin and soft tissue infection (SSI) and infection of unknown origin were the four most common foci (Table 3). Mortality was highest among patients with unknown focus (7/12). Blood cultures were obtained from all but two patients, but only 50% were positive. A culture-confirmed focus of infection was found in 73% (66/90) of patients, and 79% (71/90) had a microbiological diagnosis (based on cultures, antigen testing or PCR technique) (Table 4).

**Table 3:** Focus of infection among survivors and non-survivors

Source of infection	Frequency (%)	Survivors (*n*)	Non-survivors (*n*)	Mortality (%)	*P* value
Urinary (*n* = 33)	36.7	26	7	21.2	0.37^1^
Pulmonary (*n* = 23)	25.6	19	4	17.3	0.24^1^
Skin and soft tissue (*n* = 15)	16.7	12	3	25.0	0.52^1^
Intra-abdominal (*n* = 4)	4.4	2	2	50.0	0.29^2^
Endocarditis (*n* = 2)	2.2	1	1	50.0	0.46^2^
Others (*n* = 1)	1.1	1	0	0	0.99^2^
Unknown (*n* = 12)	13.3	5	7	58.3	*0.01* ^1^

Data are presented no. (%). ^1^Pearson chi^2^ or ^2^Fisher’s exact test. *P* values < 0.05 are shown in italics

**Table 4:** Microbiological characteristics of survivors and non-survivors

	Survivors (*n* = 66)	Non-survivors (*n* = 24)	*P* value
Positive blood culture	30 (45.5)	15 (62.5)	0.161
Any relevant culture	48 (72.7)	18 (75)	0.829
Positive diagnostics	52 (78.8)	19 (79.2)	0.969

Data are presented as no. (%) Pearson chi^2^ test. A *P* value < 0.05 was considered statistically significant. There were no signicant differences between the groups

The most common pathogen was *Escherichia coli* (*E. coli*) (23% of the patients), followed by *Streptococcus pyogenes* (10%). The frequency of *Staphylococcus aureus* (*S. aureus*) was only 7% (6/90), with a mortality of 67%, although based on very few cases. Two patients were treated for sepsis caused by extended spectrum beta lactamase-producing Enterobacteriaceae (ESBL_A_) and one patient for sepsis due to *Streptococcus pneumoniae* with reduced susceptibility to penicillin. There were no cases of methicillin-resistant *Staphylococcus aureus* (MRSA) or vancomycin-resistant Enterococci (VRE) (Table 5).

**Table 5:** Microbiological characteristics of survivors and non-survivors

Microbiological cause	Frequency (%)	Survivors (*n* = 66)	Non-survivors (*n* = 24)	Mortality %	*P* value
Gram-negative bacteria					
*Escherichia coli*	23	15	6	29	0.82^1^
*Klebsiella* spp.	6	3	2	40.	0.61^2^
Other Enterobactericeae	7	4	0	0	0.57^2^
*Pseudomonas aeruginosa*	2	2	0	0	0.99^2^
*Neisseria meningitidis*	1	1	0	0	0.99^2^
Gram-positive bacteria					
*Streptococcus pyogenes*	10	7	2	22	0.99^2^
*Streptococcus pneumoniae*	8	6	1	17	0.67^2^
Other *Streptococcus* spp.	4	3	1	25	0.99^2^
*Enterococcus.faecalis*	3	3	0	0	0.56^2^
*Staphylococcus aureus*	7	2	4	67	*0.04* ^2^
*Clostridium difficile*	1	1	0	0	0.54
Multiple pathogens	9	5	3	38	0.47
No positive diagnostics	21	14	5	26	0.97

Causative microbiological agent was based on cultures, antigen test and PCR methods. Data are presented as no. (%). ^1^Pearson chi^2^, ^2^Fisher’s exact test. *P* values < 0.05 are shown in italics

### High-risk patients

Mortality was 37% among the 46 high-risk patients. Higher age, maximum lactate level, number of failing organs and respiratory dysfunction due to infection were associated with increased mortality (Table 1).

Early appropriate antibiotic treatment and haemodynamic stability (MAP ≥ 65 mmHg) within 6 h were associated with decreased mortality. Non-survivors received more intravenous fluid in the latter part of the course of the disease (6–24 h). Limitation of level of care was more common among non-survivors (Table 2).

### Antibiotic treatment

Data on antibiotic treatment are shown in Table 2. Twenty-four out of 87 patients received the first dose of appropriate antibiotic within 1 h (data regarding three patients were missing). In all, 23% (20/87) of the patients received early appropriate antibiotic treatment including 1st and 2nd doses. In the subgroup of high-risk patients, 34% (15/44) received early appropriate antibiotic treatment.

Of the 67 patients receiving inappropriate early antibiotic treatment, the main failure was delay of the first dose in 63 cases. Major delay of the second dose was found in 13/79 patients, and nine patients had a delay in the administration of both first and second doses. The majority received cefotaxime (60%) or piperacillin-tazobactam (16%), alone or in combination with another antibiotic. Carbapenems were used in 7%. No patient received vancomycin. Of the 71 patients with known aetiology, six patients received initial treatment with an antibiotic with inappropriate spectrum.

Among the high-risk patients, administration of early appropriate antibiotic treatment correlated to a lower 28-day mortality (Table 2).

### Risk factors for inappropriate antibiotic administration

Risk factors for delay of early appropriate antibiotic treatment (shown in Table 6) were lower triage level, lower disease severity (APACHE II and SOFA score) on arrival and administration of the first dose after transfer from the ED. Patients on immunosuppression received timely antibiotic treatment more frequently.

**Table 6:** Risk factors for inappropriate antibiotic administration

	Appropriate antibiotics within 1 h *n* = 24	Appropriate antibiotics later than 1 h *n* = 63	*P* value	“Early appropriate antibiotic treatment” *n* = 20	No “early appropriate antibiotic treatment” *n* = 67	*P* value
Age (years) (SD)	73 (13)	72 (16)	0.879	71 (13)	73 (16)	0.667
Female sex (%)	12 (50)	33 (52.4)	0.843	10 (50)	35 (52.2)	0.860
Immunosuppression, any (%)	11 (45.8)	13 (20.6)	*0.019*	10 (50)	14 (20.9)	*0.011*
Inadequate empiric antibiotic(s) (%)	1 (4.2) *n* = 23	6 (9.7) *n* = 56	0.668	19 (95.0	60 (90.9)	0.999
Misdiagnosed focus of infection (%)	2 (8.3) *n* = 24	10 (16.1) *n* = 62	0.496	2 (10) *n* = 20	10 (15.2) *n* = 66	0.724
Triage level according to METTS* (SD)	1.2 (0.5) *n* = 23	2 (0.7) *n* = 57	*< 0.001*	1.2 (0.5) *n* = 19	1.9 (0.8) *n* = 61	*< 0.001*
APACHE II (SD)	22.7 (5.2)	18.4 (6.2)	*0.004*	22.1 (5.1)	18.8 (6.4)	*0.037*
SOFA score at arrival (SD)	4.8 (2.7)	3.2 (2.3)	*0.009*	4.4 (2.0)	3.4 (2.6)	0.120
Arrival with ambulance (%)	22 (95.7) *n* = 23	48 (76.2) *n* = 63	0.058	18 (94.7) *n* = 19	52 (77.6) *n* = 67	0.108
Limitation of level of care (%)	6 (25)	15 (23.8)	0.908	4 (20)	17 (25.4)	0.770
Administration of the first dose at another ward than ED (%)	1(4.2)	14 (22.2)	0.058	1 (5)	14 (20.9)	0.175

Data are presented as no. (%) or mean (SD) as indicated. The total study population and the high-risk patients are analysed separately. *t* Test, Pearson chi^2^ or Fisher’s exact test, as appropriate. *P* values < 0.05 are shown in italics

*Red triage = 1, orange triage = 2, yellow triage = 3, green triage = 4

### Achievement of treatment goals

“Achievement of treatment goal” was a secondary outcome. For all patients, an oxygen saturation > 93% within 1 h and an established MAP ≥ 65 mmHg within 6 h correlated with survival. Other treatment goals did not show any correlation with 28-day mortality, but data were missing in many cases (e.g. in 52/90 cases data were missing regarding urinary production within 6 h and in 62/90 cases regarding lactate level within 6 h).

### Multivariable analysis

A multivariable analysis was performed on the high-risk patients (Table 7). At first, we included variables with a univariate correlation to 28-day mortality (age, maximum lactate level, number of dysfunctional organs, plasma expansion at 6–24 h, early appropriate antibiotic treatment, limit of level of care and MAP ≥ 65 mmHg within 6 h) within a logistic regression. Stepwise removal of the parameter with the highest *P* value rendered a three-variable model with the parameters age, early appropriate antibiotic treatment and limit of level of care giving the best fit.

**Table 7:** 28-day mortality using multivariable analysis

Variable	OR (95% CI)	*P* value
Early appropriate antibiotic treatment	0.096 (0.011–0.846)	*0.035*
Age	1.077 (0.996–1.164)	0.062
Limitation of level of care	8.684 (1.330–56.690)	*0.024*

A logistic regression with 28-day mortality as dependent variable was performed. Stepwise removal of the parameter with the highest *P* value in the univariate analysis rendered a three-variable model with the parameters age, early appropriate antibiotic treatment and limit of level of care giving the best fit. *P* values < 0.05 were considered statistically significant in the multivariable analysis and are shown in italics. To fulfil the criterion “early appropriate antibiotic treatment,” the 1st dose must have been adequate and administered within 1 h, and the 2nd dose given with less than 25% delay after the recommended interval

The 28-day mortality was 46.4% among the high-risk patients who did not receive early appropriate antibiotic treatment compared with 12.5% among those who did (Table 2). This illustrates a more than threefold higher mortality rate among the high-risk patients who did not receive early appropriate antibiotic treatment (Table 2). In the multivariable analysis, inappropriate antibiotic therapy was found to be an independent predictor of mortality with an odds ratio for mortality of 10.4 in the high-risk group (Table 7).

## Discussion

In this retrospective cohort of adult patients with severe sepsis and septic shock, we found in the univariate analysis a greater than threefold increase in mortality rate among high-risk patients not receiving early appropriate antibiotic treatment. In the multivariable analysis, inappropriate early antibiotic therapy was found to be an independent predictor of mortality with an odds ratio for mortality of 10.4 in the high-risk group.

The importance of timing and appropriateness of the first dose of antibiotics has been shown in previous studies, especially in septic shock [9–15, 33]. Leisman et al. studied the importance of timely administration of the 2nd dose and showed that major delay in the 2nd dose was associated with higher mortality [17]. Therapeutic antibiotic concentrations in blood and at the target site must be achieved and maintained as quickly as possible in order to reduce bacterial load [20]. Thus, adequate dose and dose intervals of antibiotics are crucial for improving prognosis. In retrospective material, timing of both first and second doses may be a marker of suboptimal initial treatment and improper pharmacokinetic adaptation. In the setting of this study, antibiotic treatment was usually initiated in the ED, followed by a transfer to the ward or ICU, making delay in the second dose a risk due to logistic reasons.

The importance of adequate dosing on survival is supported by PK/PD studies [18–20] and is even more important in the treatment of critically ill patients, due to more complicated pharmacokinetics [20, 34]. Change in distribution volume influences the plasma and tissue concentrations of water-soluble antibiotics, and glomerular filtration rate may be altered [18, 35] Tissue perfusion may be decreased, making it more difficult for the antibiotic to reach its target [36]. Antibiotic blood concentrations in the critically ill are often subtherapeutic [19, 34–36], and for that reason, therapeutic drug monitoring (TDM) is becoming more widespread [34]. This stresses the importance of adequate dosage, not only the first dose but also subsequent doses of the antibiotics, in order to achieve appropriate antibiotic exposure.

In the present study, 63/87 had a delay in the first dose making this the main failure, although this did not reach statistical significance with respect to mortality in our small material. Delay in the second dose itself had only a marginal effect on mortality. Since a large proportion of patients did not receive appropriate first dose antibiotics, evaluation of the effect of a delayed second dose alone is more difficult. When looking at the combination of inappropriate first and second doses, mortality was increased more than threefold in the high-risk group. This stresses the importance of adequate antibiotic exposure based on knowledge of PK/PD. In this respect, the timing of both first and second doses may be a marker for this approach.

In order to fulfil the criteria early appropriate antibiotic treatment, the antibiotic administered must have a spectrum of activity that covers the anticipated or confirmed pathogen(s). In our series, an antibiotic with too narrow spectrum was only found in 6 of the 71 patients with a confirmed pathogen, which may be explained by the low prevalence of antibiotic resistance in Sweden.

The high-risk patients gained most benefit from early appropriate antibiotic treatment; the challenge is thus to identify these patients at an early stage. In the high-risk group, 13/27 patients who developed shock within 24 h were not identified by METTS red triage level on admission to the ED. This difficulty stresses the importance of complying with early appropriate antibiotic regimes in all cases of suspected sepsis [9, 16]. In our study, the risk factors for inappropriate antibiotic treatment were mainly related to low APACHE II score or a low SOFA score and a low METTS triage level on arrival. There is, thus, a need for improvement in the early detection of sepsis in patients with subtle symptoms. This study also illustrates the importance of prompt re-establishment of haemodynamic stability, tissue perfusion and cellular metabolism. Achievement of the SSC haemodynamic goal of a MAP ≥ 65 mmHg within 6 h was associated with increased survival. These findings are consistent with previous studies [37, 38]. Fluid therapy only differed between survivors and non-survivors in the latter part of the resuscitation period (6–24 h), where the non-survivors received more volume expansion. This probably reflects therapeutic efforts to restore haemodynamic stability in patients with prolonged circulatory failure not responding to initial fluid therapy, i.e. those with a poor prognosis. Maximum lactate level also correlated with mortality, illustrating the impact of disease severity, also seen in previous studies [39, 40].

The distribution of aetiologic pathogens and foci might have been influenced by the restricted inclusion material comprising patients from medical wards and the ICU only. *E. coli* was the most prevalent pathogen, which is in agreement with a previous study including all patients with bacteraemia in the same hospital [41]. *S. aureus*, on the other hand, was found in only six patients (7%). This incidence is lower than seen in many other studies (12–18%) based on positive blood cultures [2, 41, 42]. Our findings are supported by the National Register of Community-Acquired Sepsis of the Swedish Infectious Diseases Physicians Society, where *S. aureus* was the causing pathogen in 8–10% of cases during the same period of time [24]. The high mortality in the subgroup of *S. aureus* infections in our material (4/6) is deemed not to be representative but rather an effect of the small sample size. The prevalence of antibiotic resistance in Sweden is low, and our finding of a low level of resistant pathogens in this study is in line with Swedish resistance surveillance data [43].

Early antibiotic treatment was the main focus in this study, but data also revealed other important risk factors for mortality. Age, markers of severity of disease (maximum lactate level and number of dysfunctioning organs) and treatment (limited level of care, haemodynamic stabilisation, administration of fluids and antibiotics) were all correlated to mortality in the univariate analysis. All are known risk factors relating to outcome [39, 44–47]. The impact of treatment, including limit of level of care, is determined by the clinical management of the individual patient and thereby possible to modify in order to improve patient outcome. In the multivariable analysis, best fit was found for the model incorporating age, limited level of care and early appropriate antibiotic treatment as independent factors related to mortality. Patients with treatment limitation had, as might be expected, a higher mortality rate [46, 47]. This is an important factor to consider. Limitation of the level of care must be considered carefully since this may result in substandard care and increased morbidity and mortality if not applied correctly—fulfilment of the prophesy. In our study, those receiving limited care received more fluids in the latter part of the clinical period (6–24 h), which may have been the result of not being treated with inotropes. The mortality in the group that received limited care in our study was high, but we have no way of assessing the correctness of the restrictions applied. Whatever, care limitation must be thoroughly discussed and evaluated at the local level.

Even though our theoretical knowledge of sepsis treatment has improved over the last decade, there is much room for improvement in the practical management of the septic patient. Only 23% of patients with sepsis received early appropriate antibiotic treatment, and only 34% among obviously high-risk patients. This indicates shortcomings in the management of sepsis patients. Failure to achieve haemodynamic stability, seen as the lower number of patients with MAP > 65 mmHg at 6 h, also illustrates this point. Less than one in three patients with sepsis were treated on the ICU, even though more than half of the patients, in which treatment goals could be evaluated, were still not haemodynamically stable after 6 h.

This study has several limitations. It is a small, retrospective, single-centre study, making the results difficult to generalise. The distribution of aetiologic pathogens and infectious foci might have been influenced by the restricted inclusion material from medical wards and the ICU only. Some of the treatment goals were difficult to evaluate due to missing data.

Identification of septic patients using ICD-10 codes is also problematic and can lead to loss of cases as many patients lack specific sepsis codes at discharge [7]. Exclusion of patients who had been hospitalised within 30 days before admission also contributed to case loss.

This study also has strengths. The addition of sepsis criteria to the ICD-10 codes for inclusion resulted in a more well-defined study group. This is supported by Rhee et al., who found that sepsis based on clinical information increased the confidence in sepsis estimates, compared to identification by ICD-10 alone [5]. The use of both septic shock and METTS criteria resulted in a more precise identification of high-risk patients. Recruiting patients from medical wards and not just from the ICU better reflects the sepsis population in the Swedish healthcare system.

In conclusion, we found suboptimal initial management of septic patients at our hospital, with a more than threefold increase in mortality among high-risk patients not receiving early appropriate antibiotic treatment. Even though the importance of early antibiotic treatment has been established for a long time, adherence to this principle was very low. There is much room left for improvement in the early identification and appropriate antibiotic treatment of sepsis patients.

## Electronic supplementary material

ESM 1 (DOCX 32 kb)
