# Supplementary material for: Delay of appropriate antibiotic treatment is associated with high mortality in patients with community-onset sepsis in a Swedish setting
Source: Eur J Clin Microbiol Infect Dis. 2019 Mar 25;38(7):1223–34. doi: 10.1007/s10096-019-03529-8 (PMC6570779; doi:10.1007/s10096-019-03529-8)
Supplement: Supplementary file 1 — (DOCX 32 kb) [file 10096_2019_3529_MOESM1_ESM.docx]

**Delay of appropriate antibiotic treatment is associated with high mortality in patients with community-onset sepsis in a Swedish setting.**

**European Journal of Clinical Microbiology & Infectious Diseases**

Maria Andersson ^1^, Åse Östholm-Balkhed ^1^, Mats Fredrikson^2,3^, Martin Holmbom^1 ,4^, Anita Hällgren^1^, Sören Berg^5^, Håkan Hanberger^1^

1. Division of Infectious Diseases, Department of Clinical and Experimental Medicine, Faculty of Medicine and Health Sciences, Linköping University, Linköping, Sweden.

2. Occupational and Environmental Medicine, Department of Clinical and Experimental Medicine, Faculty of Medicine and Health Sciences, Linköping University, Linköping, Sweden.

3. Forum Östergötland, Faculty of Medicine and Health Sciences, Linköping University, Linköping, Sweden

4. Department of Urology and Department of Clinical and Experimental Medicine, Linköping University, Linköping, Sweden

5. Division of Cardiothoracic Anesthesia and Intensive Care, Department of Medicine and Health Science, Faculty of Medicine and Health Sciences, Linköping University, Linköping, Sweden.

Corresponding author

Maria Andersson,

E-mail: maria.ka.andersson@regionostergotland.se

Telephone: +46 70 533 5346

**Table 1A.** Baseline characteristics of patients without and with limits of level of care

**Total study population High risk patients**

|  | No limits of level of care (n=67) | Limits of level of care  (n=23) | P value | No limits of level of care (n=30) | Limits of level of care  (n=16) | P value |
| --- | --- | --- | --- | --- | --- | --- |
| Age, y. (SD) | 69 (15) | 84 (9) | **<0.001** | 68 (15) | 84 (9) | **<0.001** |
| Female sex (%) | 35 (52.2) | 11 (47.8) | 0.715 | 14 (46.7) | 9 (56.3) | 0.536 |
| **Preexisting comorbidity (%)** |  |  |  |  |  |  |
| Malignancy, all | 9 (13.4) | 2 (8.7) | 0.722 | 5 (16.7) | 2 (12.5) | 0.999 |
| Metastatic malignancy | 3 (4.5) | 1 (4.3) | 0.999 | 2 (6.7) | 1 (6.3) | 0.999 |
| Haematological malignancy | 4 (6) | 0 | 0.569 | 3 (10) | 0 | 0.542 |
| Diabetes mellitus | 14 (20.9) | 6 (26.1) | 0.605 | 5 (16.7) | 4 (25) | 0.698 |
| Congestive heart failure | 6 (9) | 8 (34.8) | **0.006** | 1 (3.3) | 5 (31.3) | **0.015** |
| Immunosuppression, any | 21 (31.3) | 3 (13) | 0.087 | 11 (36.7) | 2 (12.5) | 0.101 |
| Immunosuppression, >10mg Prednisolone | 8 (11.9) | 1 (4.3) | 0.438 | 4 (13.3) | 0 | 0.282 |
| Chronic pulmonary disease | 8 (11.9) | 5 (21.7) | 0.305 | 5 (16.7) | 3 (18.8) | 0.999 |
| Chronic renal failure (GFR <30) | 4 (6) | 1 (4.3) | 0.999 | 1 (3.3) | 0 | 0.999 |
| **Severity of disease** |  |  |  |  |  |  |
| APACHE II (SD) | 18.2 (6.0) | 23.6 (5.1) | **<0.001** | 21.0 (5.7) | 24.7 (5.1) | **0.035** |
| Maximum SOFA (SD) | 5.0 (3.2) | 7.0 (2.9) | **0.010** | 6.0 (3.8) | 7.8 (2.9) | **<0.001** |
| SBP<90 mm Hg / MAP<65 mm Hg (SD) | 54 (80.6) | 22 (95.7) | 0.105 | 24 (80) | 16 (100) | 0.078 |
| Septic shock (%) | 15 (22.4) | 12 (52.2) | **0.007** | 15 (50) | 12 (75) | 0.101 |
| High risk patients (%) | 30 (44.8) | 16 (69.6) | **0.040** |  |  |  |
| Maximum lactate level day 1 (SD) | 2.8 (2.1)  n=54 | 3.5 (2.6)  n=22 | 0.204 | 3.2 (2.2)  n=29 | 4.0 (2.9)  n=16 | 0.281 |
| Number of organ dysfunction (SD) | 1.3 (0.9) | 2.5 (1.3) | **<0.001** | 1.6 (1.0) | 3.0 (1.3) | **<0.001** |
| Respiratory (%) | 17 (25.4) | 16 (69.6) | **<0.001** | 12 (40) | 15 (93.8) | **<0.001** |
| Renal (%) | 38 (56.7) | 19 (82.6) | **0.026** | 21 (70) | 14 (87.5) | 0.282 |
| Haematological/coagulopathy (%) | 14 (20.9) | 8 (34.8) | 0.181 | 9 (30) | 8 (50) | 0.181 |
| CNS (%) | 12 (17.9) | 12 (52.2) | **0.001** | 7 (23.3) | 9 (56.3) | **0.026** |
| Liver (%) | 2 (3) | 2(8.7) | 0.269 | 0 | 2(12.5) | 0.116 |

| Data are presented as no. (%) or mean (SD) as indicated. The total study population  and the high risk patients are analyzed separately. T-test, Pearson Chi^2^ or Fisher’s exact test, as  appropriate. P- values <0.05 are shown in bold.  **Table 1B.** Clinical interventions, treatment evaluation and markers of awereness, based on limits of level of care  **Total study population High risk patients**   \|  \| No limits of level of care (n=67) \| Limits of level of care (n=23) \| P-value \| No limits of level of care (n=30) \| Limits of level of care (n=16) \| P-value \| \| --- \| --- \| --- \| --- \| --- \| --- \| --- \| \| Volume, *ml* , median, (IQR) \|  \|  \|  \|  \|  \|  \| \| Crystalloids 0-6h \| 2000(1000-3000 \| 3000(2000-4000) \| 0.250 \| 3000(2000-5000) \| 3000(2000-3750) \| 0.280 \| \| Plasma expansion 0-6h \| 500(250-900) \| 750(500-1050) \| 0.228 \| 950(500-1400) \| 750(500-1150) \| 0.442 \| \| Crystalloids 6-24h \| 2000(1000-3000) \| 3000(1800-3000) \| **0.015** \| 2000(1000-4000) \| 3000(2200-3500) \| 0.169 \| \| Plasma expansion 6-24h \| 500(250-910) \| 900(500-1375) \| **0.008** \| 587.5(500-1250) \| 1075(812.5-1625) \| **0.092** \| \| Appropriate antibiotics within 1h (%) \| 18 (27.7)  n=65 \| 6 (27.3)  n=22 \| 0.970 \| 13 (44.8)  n=29 \| 6 (40)  n=15 \| 0.759 \| \| 2^nd^ dose without >25 % delay (%) \| 50 (86.2)  n=58 \| 16 (76.2)  n=21 \| 0.314 \| 23 (85.2)  n=27 \| 12 (80)  n=15 \| 0.686 \| \| Early appropriate antibiotic treatment (%) \| 16 (24.6)  n=65 \| 4 (18.2)  n=22 \| 0.535 \| 11 (37.9)  n=29 \| 4 (26.7)  n=15 \| 0.455 \| \| ICU admission (%) \| 20 (29.9) \| 4 (17.4) \| 0.244 \| 13 (43.3) \| 4 (25) \| 0.220 \| \| ICU admission from ED (%) \| 8 (11.9) \| 2 (8.7) \| 0.999 \| 7 (23.3) \| 2 (12.5) \| 0.463 \| \| Within 1 h: SBP > 90 mm Hg (%) \| 42 (77.8)  n=54 \| 11 (52.4)  n=21 \| **0.030** \| 16 (59.3)  n=27 \| 5 (35.7)  n=14 \| 0.153 \| \| Within 1 h: Saturation >93 % (%) \| 41 (78.8)  n=52 \| 13 (68.4)  n=19 \| 0.365 \| 20 (76.9)  n=26 \| 7 (53.8)  n=13 \| 0.163 \| \| Within 6 hrs: Urinary production >0.5 ml/kg (%) \| 14 (50)  n=28 \| 7 (70)  n=10 \| 0.460 \| 8 (47.1)  n=17 \| 3 (60)  n=5 \| 0.999 \| \| Within 6 hrs: Lowered lactate (%) \| 12 (66.7)  n=18 \| 3 (30)  n=10 \| 0.114 \| 9 (64.3)  n=14 \| 3 (37.5)  n=8 \| 0.378 \| \| Within 6 hrs: MAP ≥65 mmHg (%) \| 30 (51.7)  n=58 \| 6 (28.6)  n=21 \| 0.068 \| 11 (44.0)  n=2 \| 3 (18.8)  n=16 \| 0.096 \| \| 28 day mortality \| 11 (16.4) \| 13 (56.5) \| **<0.001** \| 5 (16.7) \| 12 (75) \| **<0.001** \|  \|  \|  \|  \|  \|  \|  \| \| --- \| --- \| --- \| --- \| --- \| --- \| | | | | | |  |  |  |  |  |
| --- | --- | --- | --- | --- | --- | --- | --- | --- | --- | --- | --- | --- | --- | --- | --- | --- | --- | --- | --- | --- | --- | --- | --- | --- | --- | --- | --- | --- | --- | --- | --- | --- | --- | --- | --- | --- | --- | --- | --- | --- | --- | --- | --- | --- | --- | --- | --- | --- | --- | --- | --- | --- | --- | --- | --- | --- | --- | --- | --- | --- | --- | --- | --- | --- | --- | --- | --- | --- | --- | --- | --- | --- | --- | --- | --- | --- | --- | --- | --- | --- | --- | --- | --- | --- | --- | --- | --- | --- | --- | --- | --- | --- | --- | --- | --- | --- | --- | --- | --- | --- | --- | --- | --- | --- | --- | --- | --- | --- | --- | --- | --- | --- | --- | --- | --- | --- | --- | --- | --- | --- | --- | --- | --- | --- | --- | --- | --- | --- | --- | --- | --- | --- | --- | --- | --- |
| Data are presented as no. (%) or mean (SD) unless otherwise indicated. The total study population and the high  risk patients are analyzed separately. T-test, Pearson Chi^2^ or Fisher’s exact test, as appropriate. Volume  calculated with Mann-Withney U. P- values <0.05 are shown in bold.  Abbreviations: SBP systolic blood pressure, MAP mean arterial pressure, BE Base Excess, SD standard  deviation, ICU intensive care unit, SOFA sequential organ failure assessment, APACHE acute physiology and  chronic health evaluation, ED emergency department |  |  |  |  |  |  |  |  |  |  |
